# Cerebral Venous Thrombosis in Paroxysmal Nocturnal Hemoglobinuria: A Series of 15 Cases and Review of the Literature

**DOI:** 10.1097/MD.0000000000000362

**Published:** 2015-01-09

**Authors:** Elodie Meppiel, Isabelle Crassard, Régis Peffault de Latour, Sophie de Guibert, Louis Terriou, Hugues Chabriat, Gérard Socié, Marie-Germaine Bousser

**Affiliations:** From the AP-HP, Hôpital Lariboisière (EM, IC, HC, M-GB), Service de Neurologie; AP-HP, Hôpital Saint Louis (RPL, GS), Service d’Hématologie Greffe, Paris; Centre Hospitalier Pontchaillou (SG), Service d’Hématologie Clinique, Rennes; Hôpital Claude Huriez (LT), Service des Maladies du Sang, Lille; and INSERM 728 (GS), Université Paris 7 Denis Diderot, Paris, France.

## Abstract

Paroxysmal nocturnal hemoglobinuria (PNH) is a rare acquired disorder of hematopoietic stem cells characterized by hemolytic anemia, marrow failure, and a high incidence of life-threatening venous thrombosis. Cerebral venous system is the second most frequent location of thrombosis after hepatic veins. However, data about PNH-related cerebral venous thrombosis (CVT) are very scarce because of the rarity of both the disorders.

We report a French study about PNH patients with CVT. Patients were recruited retrospectively, from the Société Française d’Hématologie (SFH) registry of 465 patients with PNH; the Lariboisière registry of 399 patients with CVT; and a direct contact with 26 French Hematology Units. We review cases reported since 1938 in the English and French language literature. We then compared patients of our series with cases from the literature, with non-PNH-related CVT cases from Lariboisière registry, and with PNH patients without CVT from SFH registry.

Fifteen patients were included between 1990 and 2012. Most patients were women (12/15) and half of them presented associated hormonal venous thrombosis risk factors. Three patients had concomitant hepatic vein thrombosis. CVT was the first manifestation of PNH in 4 patients. No major difference in CVT characteristics was found compared with non-PNH-related CVT cases, except for a younger age at diagnosis in PNH patients (*P* < 0.001). All patients were treated with anticoagulation therapy. One death occurred in acute stage. All surviving patients were independent 1 year after. Median survival time was 9 years. Recurrent thrombosis rate was 50% at 6 years, occurring in patients that did not have bone marrow transplantation or eculizumab therapy. Cases of death were mainly related to hepatic vein thrombosis.

Prognosis of CVT was good in our series. However, these patients have a poor long-term prognosis due to PNH disease by itself. PNH treatment should be proposed as soon as possible to avoid recurrent thrombosis. Besides, inaugural CVT events encourage investigating PNH in case of cytopenia, hemolysis, abdominal veins thrombosis, or aplastic anemia history associated with the neurological complication.

## Abstract 2

Supplemental Digital Content is available in the text

## INTRODUCTION

Cerebral venous thrombosis (CVT) is an infrequent variety of stroke. Its incidence in adults is estimated at 1.32 per 100,000 person-years, with a higher rate among women and middle-aged patients.^1^ CVT presents with a wide spectrum of symptoms and modes of onset. Magnetic resonance imaging (MRI) with magnetic resonance angiography (MRA) are the best neuroimaging techniques for diagnosis and heparin is the first-line therapy.^2^ The outcome is often favorable with a mortality between 3% and 8% in recent studies.^1,3^

Paroxysmal nocturnal hemoglobinuria (PNH), an acquired disorder of hematopoietic stem cells, is an extremely rare cause of CVT. It is characterized by hemolytic anemia, bone marrow failure, and episodes of thrombotic events. According to PNH studies,^4–10^ venous thrombosis occur in up to 40% of patients and represent the major cause of death. The location of thrombosis is remarkably unusual, involving mostly abdominal and cerebral veins. CVT are reported in 2% to 8% of patients with PNH.^4–10^

However, data about CVT in PNH are very scarce. Only 32 patients have been reported since 1938, mostly as single case reports^11–38^ or series of 2 patients.^39,40^ No study has so far addressed the specific characteristics of this rare association. The aim of the present study was to report a series of patients with PNH-related CVT, compare these patients to patients with CVT but no PNH, compare these patients to patients with PNH but no thrombosis or noncerebral thrombosis, and compare our series to cases of PNH-related CVT reported in the literature.

## METHODS

### Patients

The patients of the PNH-CVT series were recruited in 2 steps. First, we sought cases among 2 consecutives registries. The Société Française d’Hématologie (SFH) registry of 465 patients with PNH, included between 1950 and 2005.^10^ We considered patients after 1990 (n = 340) to ensure reliable neuroradiological data. We reviewed medical records of patients who had a stroke and included those with CVT. The prospective Lariboisière registry that included 399 patients with CVT between 1997 and 2012. In a second step, we contacted French Hematology Units by electronic mailing to inquire about cases of PNH-related CVT occurring after 2005.

The patients should meet the following criteria to be included in this study: PNH diagnosis based on flow cytometry analysis or Ham test; CVT diagnosis based on relevant neuroimaging criteria, meaning the presence of typical signal abnormalities of the thrombus on MRI (T1, T2-weight images, or T2 gradient echo) and/or the absence of flow in the occluded sinuses or veins on MRA, computed tomography angiography, or digital subtraction angiography; sufficient data about PNH history (date of diagnosis, history of thrombosis, and ongoing treatment); clinical features at the time of CVT (type of symptoms and modes of onset, and outcome at discharge); and follow-up (date and major events since CVT).

Data were collected directly from the patient's medical record. The date of PNH diagnosis was based on the first positive flow cytometry analysis or Ham test. The classification in aplastic anemia (AA)-PNH required the aplasia criteria^10^: presence of at least 2 or 3 blood cytopenia (hemoglobin level <10 g/dL; platelets < 80 × 10^9^ cells/L; neutrophil < 1 × 10^9^ cells/L). In case of past venous thrombotic events, location and date of occurrence were reviewed. At the time of CVT, the following items were recorded: level of hemoglobin, platelets and neutrophil; PNH and/or anticoagulant treatment; associated venous risk factor and thrombophilia checkup, if realized; and concomitant venous thrombosis. Clinical signs of CVT were recorded, with the mode of onset defined as the time between first symptoms and the diagnosis: acute (<2 days), subacute (2–30 days), and chronic (>30 days). Neuroimaging findings were collected by specifying the location of the thrombus in the cerebral veins and/or sinus and the presence of a parenchymal injury. Type of treatment and occurrence of worsening at the acute phase were analyzed. Disability at discharge and at 1 year was classified according to the modified Rankin scale (mRS) as good recovery (mRS 0–1); partial recovery (mRS 2); partial dependency (3–4); total dependency (mRS 5); and death (mRS 6). Specific management for PNH after CVT such as bone marrow transplant (BMT) or eculizumab was recorded. Last, data about follow-up were collected: occurrence of new thrombosis, date of death, and cause.

### Literature Review

For the literature review, a Medline search was performed using the following terms: “cerebral venous thrombosis,” “cerebral sinus thrombosis,” “paroxysmal nocturnal hemoglobinuria,” and “Marchiafava-Micheli disease.” We selected cases published in full papers (excluding abstracts) in English and French language literature since 1938. References of selected publications were hand searched for further titles. In addition, cases of PNH were searched from the CVT series published since 1990. Inclusion criteria were patients with an acute proven CVT using neuroimaging and/or postmortem examination, PNH diagnosis based on flow cytometry analysis or Ham Test, available clinical data concerning PNH history (date of diagnosis and thrombosis history), CVT symptoms at onset, location of the thrombus, and presence of a parenchymal injury, type of treatment, and outcome at discharge (good recovery and death). We collected data about associated venous risk factors and concomitant venous thrombosis if available.

### Statistical Analysis

Three comparisons were made. We compared our PNH-CVT series first to PNH-CVT cases published after 1990, second to CVT patients with no PNH from the prospective Lariboisière Registry, and third to PNH patients from the SFH Registry who did not have thrombosis (n = 272) or did have a noncerebral thrombosis (n = 56). The absence of overlapping cases between different groups was verified. Comparisons were performed using the χ^2^ test or Fisher exact test when necessary, for the dichotomous data, and the Student *t* test for continuous data. Survival rates were estimated by the Kaplan–Meier method. Survival curves were compared using a Mantel–Byar approach. PNH patients with CVT had follow-up starting from the date of CVT, PNH patients with noncerebral thrombosis starting from the date of noncerebral thrombosis, and PNH patients without thrombosis starting from PNH diagnosis, in order to avoid time-dependent bias. We then added the follow-up of patients with thrombosis (whether cerebrovascular or not) between PNH diagnosis and thrombosis to the group of patients without thrombosis, according to the Mantel–Byar approach. We have added a Cox Proportional Hazards analysis adjusted for age and gender. A probability value <0.05 was used to define statistical significance.

## RESULTS

### PNH-CVT Series

Fifteen PNH patients with CVT were included, 8 from the SFH Registry (out of 340 patients between 1990 and 2005), including 3 coming from the Lariboisière CVT Registry and 7 from 26 French Hematology Units after 2005 (out of 27 that were contacted). Among the SFH Registry, 4 more patients were reported as having had CVT but were excluded because of missing neuroradiological data.

Baseline characteristics of patients and PNH are detailed in Table 1 and synthesized in Table 2. A large majority of patients were women, half of whom had hormonal thrombotic factors. Eight patients had AA-PNH at diagnosis, and blood count cells at the time of CVT fulfilled aplasia criteria for 10. Two patients had a previous hepatic vein thrombosis 1 year before CVT and were treated by anticoagulant therapy when CVT occurred, respectively, with low-molecular-weight heparin (LMWH) and danaparoid. Other ongoing hematological treatment is described in Table 1. CVT revealed PNH in 4 patients, and in 3 of them, hematological data were available and suggested PNH diagnosis: anemia and thrombocytopenia in all, elevated level of lactate dehydrogenase, decreased level of haptoglobin and hemoglobinuria in 2, and history of AA in 2. Among these 3 patients, PNH diagnosis was overlooked in 1 and established 1 year after when he had hepatic vein thrombosis.

**TABLE 1:**
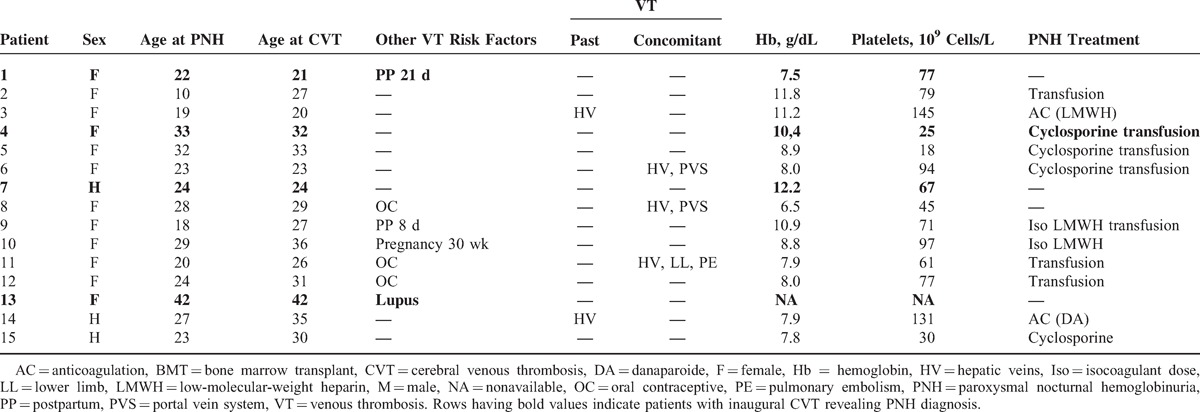
Baseline Characteristics of Patients With PNH at the Time of CVT in PNH-CVT Series (n = 15)

**TABLE 2:**
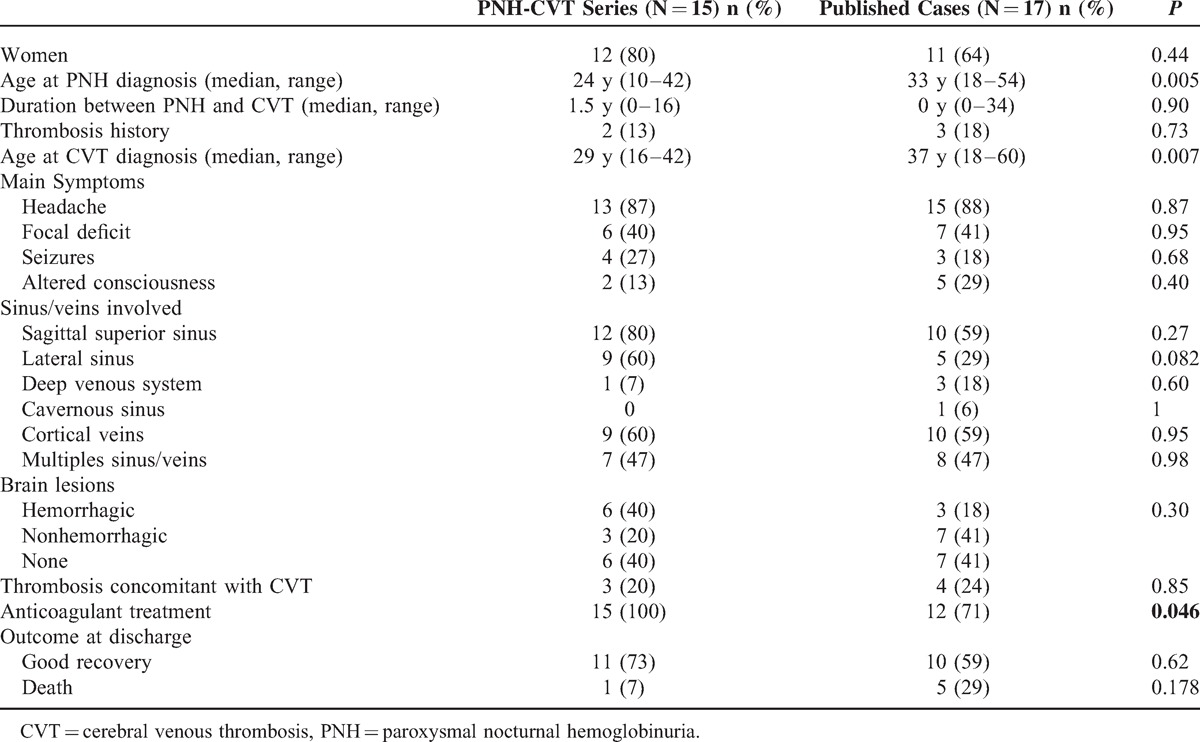
CVT in PNH: PNH-CVT Series and Published Cases (>1990)

CVT characteristics are detailed in Table 3 and synthesized in Table 2. Headache was the most common symptom and was isolated in 3 patients. Clinical onset of CVT was subacute in two-third of patients (10/15). Other symptoms were focal deficits, seizures, and less frequently altered consciousness. MRI was performed in almost all patients (14/15) and was the first investigation in 11. Location of thrombosis and brain lesions are exposed in Tables 2 and 3. Three patients had extra neurological venous thrombosis, 3 had hepatic and/or portal vein thrombosis, while 1 had pulmonary embolism. Anticoagulation was started on the day of CVT diagnosis in all patients: unfractionated heparin (UH) in 10, danaparoide in 3, and LMWH in 1. Oral contraceptive pills were stopped. Eleven patients improved rapidly but 4 deteriorated within the first days of treatment, with worsening of their neurological deficit, intracranial hypertension, and/or consciousness. Three improved after neurological and hematological therapeutic adjustments (modification of the type of anticoagulant treatment in all, symptomatic treatment of intracranial hypertension in 1, and adaptation of antiepileptic treatment in 1). One patient died despite heparin therapy adjustment and mechanical thrombectomy. At discharge, 11 patients had no disability (mRS 0–1), 1 had partial recovery (mRS 2), and 2 were partially dependent (mRS 3–4). At 1 year, all patients were independent (mRS 0–2).

**TABLE 3:**
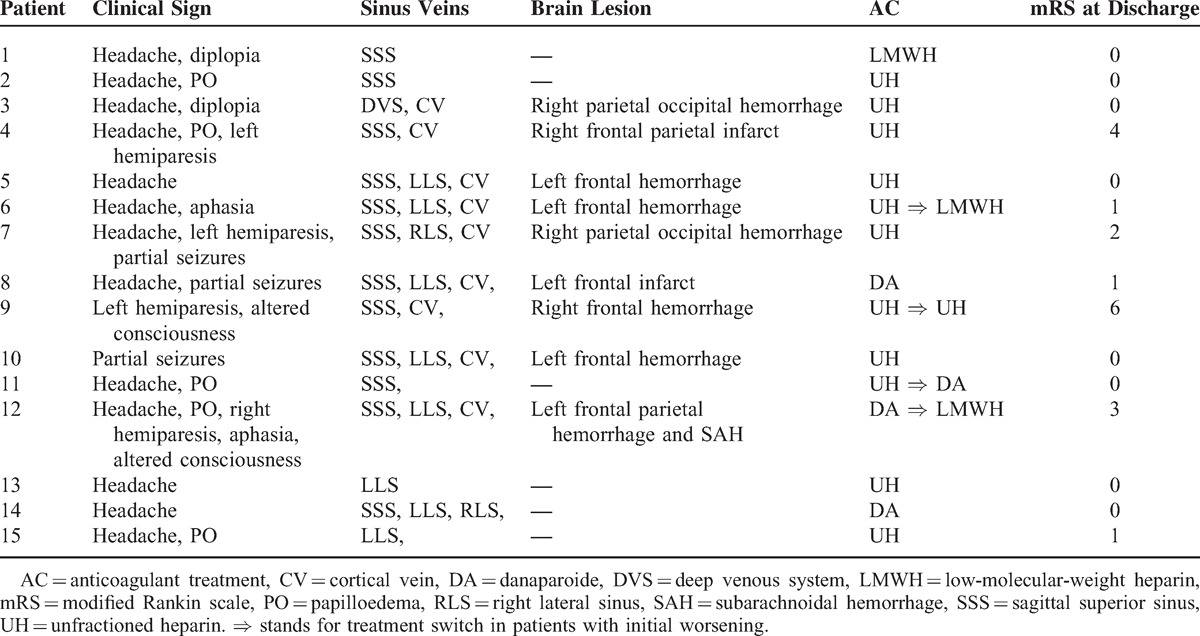
CVT Characteristics in PNH-CVT Series (n = 15)

PNH management after CVT occurrence showed substantial differences between the patients. In 7 patients, treatment was based solely on long-term anticoagulation. During a median follow-up of 6 years (range 1.3–18), 5 patients experienced another thrombosis located in hepatic and/or portal veins. Three patients died of Budd–Chiari syndrome and 1 of infection. Seven other patients received a specific PNH treatment: BMT 4 to 7 months after CVT in 4 patients, and eculizumab 1 to 60 days after CVT in 3 patients. Median follow-up was 4.7 years (range 0.2–10). No patient had recurrent thrombosis. Two deaths occurred, related to graft versus host reaction in one and to an unknown cause in the other.

**FIGURE 1:**
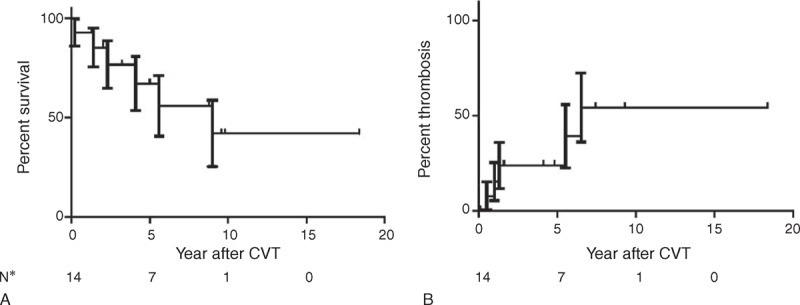
(A) Overall survival and (B) thrombosis recurrence rate in the 15 patients of PNH-CVT series. CVT = cerebral venous thrombosis, PNH = paroxysmal nocturnal hemoglobinuria.

On follow-up after CVT, the overall median survival time was 9 years (Figure 1A) and the recurrent thrombosis rate was 50% at 6 years (Figure 1B). None of the recurrent thrombosis was a CVT. One patient experienced a complicated pregnancy few years after CVT, with major hemolytic crisis and spontaneous abortion.

### Literature Review

Scott et al^11^ reported the first case of patient with PNH and CVT in 1938. Since then, 31 other cases have been published in the English and French language literature.^12–40^ From the published CVT series since 1990,^3,41–45^ only 1 case of PNH was clearly identified^41^ but could not be included because of insufficient clinical data.

Sixty-nine percent of CVT-PNH patients in literature were women (22/32). Median age at CVT was 35 (range 18–66). Associated risk factors for venous thrombosis were reported in 5 patients: oral contraceptives in 2, postpartum period in 2, and meningitis in 1. The majority of patients had headache (26/32), half of them had focal deficits (17/32), and a third had altered consciousness (11/32) at the time of CVT diagnosis. The superior sagittal sinus (SSS) was the most common site of thrombosis (20/32). A parenchymal lesion was reported in two thirds of patients (21/32), and hemorrhagic in half of them. Forty percent of patients (12/32) had another location of venous thrombosis involving preferentially abdominal veins but some had also arterial events, such as ischemic stroke in 3 or myocardial infarction in 1. Five patients had a previous venous thrombosis but none was anticoagulated when CVT occurred. CVT was inaugural in 10 patients. In 3, PNH was diagnosed several months after CVT. The overall mortality rate was 15/32 (47%), 4 (80%) before 1970, 6 (60%) between 1970 and 1990, and 5 (29%) after 1990.

### Comparisons

The comparison between PNH-CVT series and cases published after 1990 is summarized in Table 2. Except for a younger age, CVT clinical and radiological characteristics are similar.

The comparison between PNH-CVT series and the 396 non-PNH-related CVT from the Lariboisière Registry (Supplemental Table 1, http://links.lww.com/MD/A128, that exposes the comparison of demographic data and main CVT characteristics between the 2 groups) shows a younger age (median age 29 vs 35, *P* < 0.001) in PNH-CVT. CVT characteristics are similar except for a higher frequency of SSS involvement and a lower frequency of right lateral sinus involvement in PNH-CVT, 80% versus 45% (*P* < 0.05) and 13% versus 46% (*P* < 0.05), respectively. The outcome at discharge is broadly similar in both the groups, with a good recovery in 73% and 70% and a death rate of 7% versus 2%.

**FIGURE 2:**
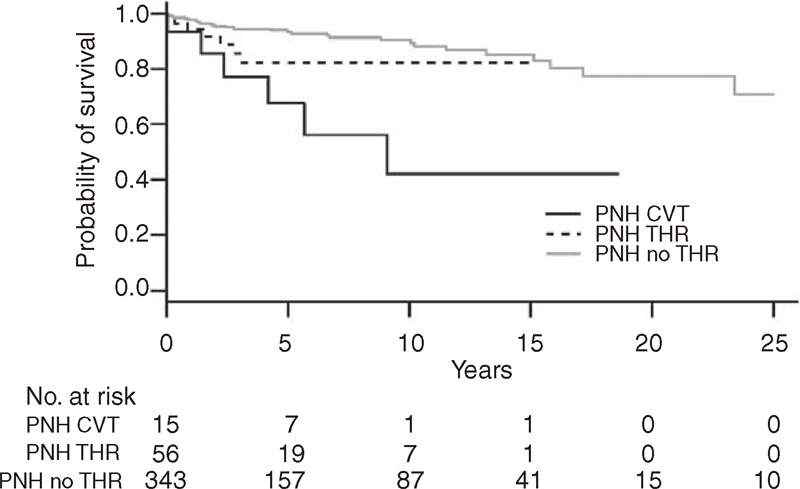
Survival analysis in paroxysmal nocturnal hemoglobinuria: survival in patients with cerebral venous thrombosis (n = 15, present series), in patients with other thrombosis (n = 56, from the Société Française d’Hématologie registry), and in patients without thrombosis (n = 272, from the Société Française d’Hématologie registry). CVT = cerebral venous thrombosis, PNH = paroxysmal nocturnal hemoglobinuria.

Among the 328 PNH patients without CVT of the SFH Registry, almost 20% (56/328) had thrombosis in noncerebral locations: abdominal veins in 50, lower limbs in 17, and pulmonary embolism in 5. We compared the patients of PNH-CVT series with the patients of the SFH Registry as exposed in the Supplemental Table 2, http://links.lww.com/MD/A128, setting out the analysis in 2 steps: comparison of PNH-CVT first with the 272 PNH patients with no thrombosis, and second with the 56 PNH with noncerebral venous thrombosis. Compared to patients with no thrombosis, patients with CVT, younger at PNH diagnosis (median age of 24 vs 33, *P* < 0.001), have a higher female preponderance (80% vs 51%, *P* < 0.05). Proportion of AA-PNH was similar in both the groups. Compared to patients with noncerebral thrombosis, patients with CVT are younger at the time of PNH diagnosis (median age 24 vs 37, *P* < 0.05) and at the time of thrombosis (median age 29 vs 40, *P* < 0.005). The proportion of women is not statistically different (80% vs 57%, *P* = 0.14). Survival curves of the 3 groups are exposed in Figure 2. CVT occurrence is associated with a statistically significant lower survival rate, compared to patients with no thrombosis and also to patients with noncerebral thrombosis after adjustment on age and sex (Table 4).

**TABLE 4:**
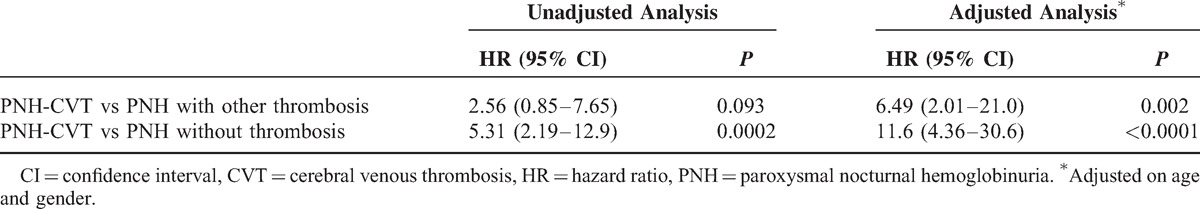
Unadjusted and Adjusted Analysis of Overall Survival, According to Mantel–Byar Approach: Comparison of Patients With CVT (n = 15, PNH-CVT Series) to Patients With Other Thrombosis (n = 56, From the SFH Registry) and to Patients Without Thrombosis (n = 272, From the SFH Registry)

## DISCUSSION

We report the largest series so far of PNH-related CVT, including 15 patients. This series confirms that PNH is a very rare cause of CVT, 0.8% in our large prospective CVT Registry. The frequency of CVT in the SFH PNH Registry is 2% to 3% in keeping with the 2% to 8% published rates in PNH patients,^4–10^ and cerebral location ranks third, accounting for 14% of all thrombosis. The figure (2%–3%) in our series is most likely an underestimation given the presence in the PNH SFH Registry, first of 4 cases labeled as CVT but not included in the present study because of missing neuroradiological data, and second of 9 cases of intracranial hemorrhage of unknown cause that might have been because of nonrecognized CVT.

The comparison of our series with the 17 PNH CVT published since 1990 is difficult because of the greater heterogeneity of the reported cases. Nevertheless, except for a younger age and more frequent anticoagulation in our patients, our data are in accordance with the previous data. A major point is the 30% frequency of other venous thromboembolic events either in the past or concomitant with CVT that is much higher than the rate observed in CVT because of other causes: it was 4% in the International Study on Cerebral Veins and Dural Sinus Thrombosis.^3^ Furthermore, the veins affected are mostly abdominal (hepatic veins or portal system) that is extremely rare in CVT related to other causes.

Another important point is that CVT may occur anytime in the course of PNH: it may reveal the disease or occur decades after PNH diagnosis. Both in our series and in published cases, CVT revealed PNH in about 30% of cases. Since CVT itself has no specific characteristics, it is on the basis of systematic clinical examination and routine laboratory tests that suspicion of PNH should be raised: episodes of hemoglobinuria, history of idiopathic AA, anemia, thrombocytopenia, biological signs of hemolysis, and past or present venous thromboembolic events should point to the diagnosis and performed flow cytometry.

A third point is that the outcome of CVT is traditionally worse in PNH-related CVT than in other causes of CVT. We found no difference between our 15 cases and other CVT from our prospective CVT Registry with regard clinical presentation, frequency of hemorrhagic brain lesion, and rates of good recovery, around 70% at discharge in both the groups. However, this was obtained after an initial deterioration requiring modification of the type of anticoagulation and neurological treatment in 25% of patients. UH has been suspected to be detrimental in PNH patients^46–48^ without any formal proof. Indeed, in the acute stage of CVT, the prompt initiation of a specific PNH therapy should be discussed, especially eculizumab that lowered the rate of thrombotic events in association with anticoagulation.^49^ Death rates, although not statistically different are, however, divergent: 2% in non-PNH-CVT from our CVT Registry, 7% in PNH-CVT in the present series, and 29% in recent published cases of PNH-CVT. The exact cause of death being difficult to assert in many cases, it is impossible to know whether this higher death rate in PNH patients is related to a more severe CVT or another cause of death such as pulmonary embolism. It thus seems that although more difficult to treat initially, and with possibly a slightly higher death rate, the prognosis of PNH-related CVT have the same good functional prognosis as other CVT.

A fourth point illustrated by our series and the review of published cases is the marked preponderance in young women. The mean 72% rate of women is about 10% higher than in other sites of venous thrombosis in PNH patients: 60% in the PNH SFH Registry and in the European cohort of PNH patients with Budd–Chiari syndrom.^50^ Similarly, PNH patients with CVT are 5 to 10 years younger than both other CVT and PNH patients. The role of hormonal risk factors is well established for CVT, in general, either in isolation or in association with another cause or risk factor.^3^ This holds true for PNH; half of our patients had hormonal risk factors, including postpartum that carries a particularly high risk in PNH as illustrated in a recent study of 25 patients in postpartum.^51^ This well-known multifactorial pattern of CVT emphasizes the need for a detailed etiological work-up even in patients who have an obvious risk factor such as postpartum state or an obvious cause such as PNH.

One of the major points of this study is the poor long-term prognosis of PNH patients after CVT, mainly related with new venous thrombotic events. In most cases, anticoagulation treatment is insufficient to prevent thrombosis recurrence.^10,52,53^ Specific hematological support, immediately after neurological episode, seems decisive for the prognosis. In the present series, patients who received allogeneic BMT or eculizumab treatment had a lower mortality rate and no recurrent thrombosis. However, our recent European PNH survey strongly questioned the benefit of allogeneic BMT in the context of thrombosis.^54^ The survival was significantly worse for patients transplanted as compared with nontransplanted patients. These patients were, however, transplanted before the use of eculizumab. Our study highlights that patients with CVT have a particularly unfavorable long-term survival, compared to patients with no thrombosis but also to patients with noncerebral thrombosis. This result suggests that cerebral location of thrombosis might be a marker of severity of PNH disease.

Our study did not analyze the mechanisms leading to thrombosis in PNH, not yet fully understood. A recent review highlighted that the complement and coagulation systems are closely integrated with each, influencing the activity of the other.^55^ Proportion of CVT in classical and AA-PNH was the same in our study, concordant with the SFH study of Peffault et al^54^ highlighting a relatively close incidence of thrombosis in both forms.

Limitations of the present study are the retrospective nature of recruitment and the small number of patients. They exposed mainly to selection bias that could underestimate the prevalence and the severity of CVT in PNH patients. Indeed, the study considered only diagnosed and treated CVT in PNH patients while we know that some cases of CVT could lead to an acute neurological impairment with a fatal issue, before an accurate diagnosis could be established.

In conclusion, PNH-related CVT are rare, with no specific characteristics except for a marked preponderance in young females, a frequent association with past or concomitant abdominal vein thrombosis, and a more complex initial therapeutic approach, requiring a close collaboration between neurologists and hematologists. CVT may reveal PNH that should thus be considered in the presence of some clinical or biological warning signs, even in the presence of other causes or risk factors for venous thrombosis such as hormonal risk factors. While the prognosis of CVT is good in our series, PNH evolution is characterized by a poor long-term survival. It was related mostly to a high rate of recurrent venous thromboembolic events, particularly in the absence of specific hematological treatment. However, the efficacy of various specific therapeutic approaches particularly BMT is debated and required to be assessed in large long-term study.
